# Jean Jacques Lhermitte (1877–1959)

**DOI:** 10.1007/s00415-018-9101-7

**Published:** 2018-10-30

**Authors:** Andrzej Grzybowski, Marta Pugaczewska, Aleksandra Pięta, Igor Stolarek

**Affiliations:** 1 Institute for Research in Ophthalmology, Fundacja Okulistyka 21, Poznan, Poland; 2 Department of Ophthalmology, University of Warmia and Mazury, Olsztyn, Poland; 3 Faculty of Medical Science, University of Warmia and Mazury, Olsztyn, Poland; 4 Faculty of Veterinary Medicine, University of Warmia and Mazury, Olsztyn, Poland

Jean Lhermitte was a renowned French neurologist and psychiatrist, whose name can be found in many medical eponyms, including the Lhermitte’s sign observed in multiple sclerosis patients.

Jean Lhermitte was born on 30 January 1877 in Mont-Saint-Père in Champagne, son of Léon Augustin Lhermitte, a French realist painter, and Heloise Goudard. His younger brother Charles Augustin was a photographer. Famous artists Auguste Rodin (1840–1917) and Vincent van Gogh (1853–1890) were friends of the family. Following his early education at Saint-Étienne school in Meaux, he studied in Paris, where he attended clinical classes (*externat*) in 1896 and worked as a resident (*internat*) in 1900. Between 1905 and 1906 he was the deputy of Fulgence Raymond (1844–1910) at the Pathological Anatomy Laboratory. In 1907, he defended his doctoral thesis entitled *Étude sur les paraplégies des viellards* (Studies on paraplegia in the elderly) and graduated with honours from the Faculty of Medicine in Paris. A year later, he was appointed the director of the neurological clinic at l’Hôpital de la Salpêtrière alongside Professor Raymond. In 1910, he was appointed the head of Pierre Marie’s (1853–1940) laboratory, where, in cooperation with neuropathologist Gustav Roussy, he conducted research on pathological anatomy and histological changes in the nervous system [1]. During this period his scientific work was less intense due to his wife’s illness.

After the outbreak of World War I in 1914, Lhermitte was assigned to an infantry regiment as a field doctor, and a year later he started working with Henri Claude (1869–1945) at the neurological centre of the 8th military region in Bourges, where he conducted research on traumatic spinal cord injuries and neuroendocrine disorders [2]. He was also interested in neuropsychiatric disorders among war victims and veterans [3]. In 1919, he was appointed director of the Paul-Brousse hospital in Villejuif.

In 1923, Lhermitte received the title of *professeur agrégé* of psychiatry and took up a position at the Department of Mental and Neurological Diseases in Paris headed by Henri Claude. Despite his scientific achievements in the field of neurology, Jean Lhermitte was never awarded the formal title of professor of neurology because the only existing position in Paris at the time of his professional activity was already filled.

In 1928, Jean Lhermitte became the head of the Dejerine Laboratory, where he worked until its closure in 1945. After the outbreak of World War II, he again took care of the sick at the Paul-Brousse hospital. In 1944, after the disappearance of Professor Joseph Lévy-Valensi (1879–1943), he was offered the chair of psychiatry, but not knowing what happened to the professor and in the absence of official information about his death, he nobly refused. A few years later, the age limit prevented him from taking up the position and instead, he was appointed *professeur honoraire* in 1948. In 1947, he had his first heart attack. Despite his state of health, he continued his scientific work with passion and commitment. In the summer of 1957 he suffered a second heart attack, which eventually forced him to take a rest and partly withdraw from his scientific career. Jean Lhermitte died on 2 January 1959 in his sleep at the age of 81. He is buried in a family grave in the Aisne region in France [4]. In 1907, Lhermitte married Lucie Megret, but was widowed in 1916 and in 1918 married Marcelle Duflocq. The couple had four children. Their son François Lhermitte (1921–1998) was a professor of neurology and neuropsychiatry at the Faculty of Medicine in Paris.

Jean Lhermitte was the author of more than 800 scientific papers and 16 books. His areas of interest included neurology, neuropathology, psychiatry, psychology and even mystical phenomena, as he also conducted research on demoniacal possession and stigmata. In 1920, together with Paul Duclos, he was the first to describe dysplastic cerebellar gangliocytoma, a rare, benign tumor of the cerebellum of partly unknown etiology, occurring more frequently in men in the second and third decade of life, which is now called the Lhermitte–Duclos disease [5].

He also described internuclear ophthalmoplegia, which results from a lesion of the medial longitudinal fasciculus connecting the nucleus of the abducens nerve with the motor nucleus of the oculomotor nerve (Lhermitte’s syndrome). The disorder manifests itself by the inability to adduct the eyeball on the side of the damage with simultaneous occurrence of nystagmus in the abducted eye. Together with Douglas McAlpine, he described a set of symptoms characteristic of pyramidal and extrapyramidal system damage occurring in the elderly as a result of atherosclerotic lesions (Lhermitte-McAlpine syndrome). Characteristic symptoms are muscle stiffness, bradykinesia, as well as facial, throat and laryngeal muscle disorders. Apathy, hallucinations, delirium, and disorientation may also occur [6].

He described a symptom in multiple sclerosis patients which consisted in a sensation of a 2-s distal electrical discharge that runs along the spine and limbs in response to an active or passive forward bending of the head. Although Pierre Marie and Charles Chatelin [7] made the first description of this symptom in 1917, it was Lhermitte who first took notice of the importance of its occurrence as an early sign of multiple sclerosis and suggested its close connection with the demyelination of the posterior spinal cord. He also proposed possible causes of this phenomenon other than multiple sclerosis [8, 9].

He was the first to describe a syndrome of hallucinations, seen originally in a 72-year-old female patient who saw life-like images of small animals and humanoid creatures, as well as children playing, mainly at dusk, and with partial awareness of their unreality. On the basis of neurological examination, he proposed the location of the brain damage in midbrain and pons [10]. This neuropsychiatric disorder is now called Lhermitte’s peduncular hallucinosis.

**Fig. 1:**
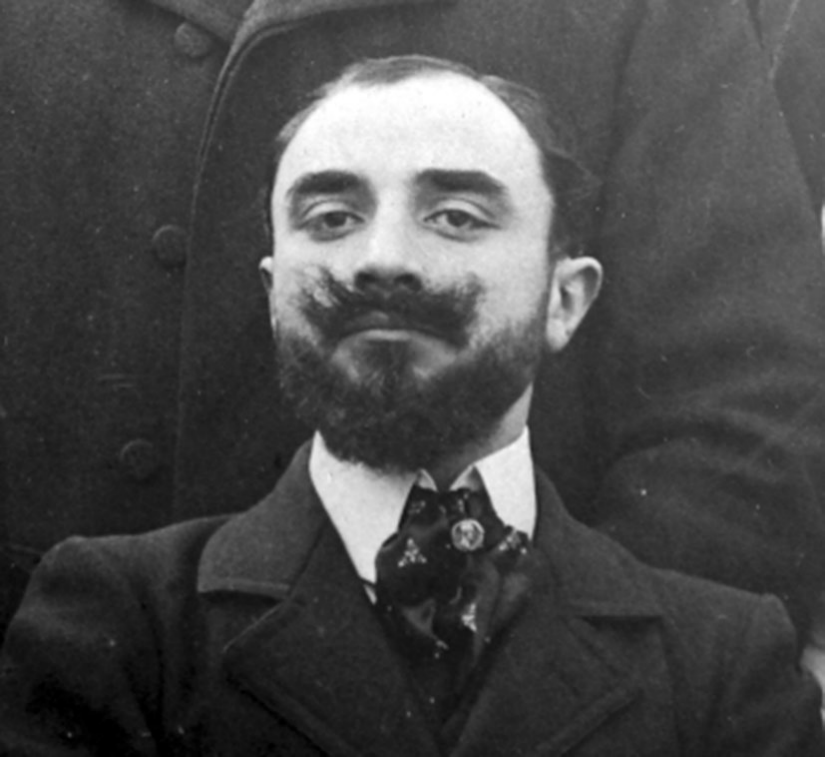
Jean Lhermitte as an intern at La Salpêtrière hospital in 1901. Public domain. Source: https://en.wikipedia.org/wiki/Jean_Lhermitte#/media/File:Lhermitte_1901.gif

In collaboration with Pierre Marie, he presented an early description of the pathology of Huntington’s disease. He also worked on olivopontocerebellar atrophy, pathogenesis of cerebrovascular diseases, phantom limb pain and many other pathologies of the central and peripheral nervous system, as well as mental disorders (Fig. 1).
